# PTSD is associated with neuroimmune suppression: evidence from PET imaging and postmortem transcriptomic studies

**DOI:** 10.1038/s41467-020-15930-5

**Published:** 2020-05-12

**Authors:** Shivani Bhatt, Ansel T. Hillmer, Matthew J. Girgenti, Aleksandra Rusowicz, Michael Kapinos, Nabeel Nabulsi, Yiyun Huang, David Matuskey, Gustavo A. Angarita, Irina Esterlis, Margaret T. Davis, Steven M. Southwick, Matthew J. Friedman, Traumatic Stress Brain Study Group, Matthew J. Girgenti, Matthew J. Friedman, Ronald S. Duman, John H. Krystal, Ronald S. Duman, Richard E. Carson, John H. Krystal, Robert H. Pietrzak, Kelly P. Cosgrove

**Affiliations:** 1 Interdepartmental Neuroscience Program, Yale University, New Haven, CT 06510 USA; 2 Department of Radiology and Biomedical Imaging, Yale School of Medicine, New Haven, CT 06520 USA; 3 Department of Psychiatry, Yale School of Medicine, New Haven, CT 06511 USA; 4 Yale PET Center, Yale School of Medicine, New Haven, CT 06519 USA; 5 U.S. Department of Veterans Affairs, National Center for Posttraumatic Stress Disorder, Clinical Neurosciences Division, VA Connecticut Healthcare System, West Haven, CT 06516 USA; 6 Neurosciences Center, Dartmouth Medical School, Hanover, NH 03755 USA

**Keywords:** Post-traumatic stress disorder, Microglia, Molecular neuroscience, Neuroimmunology, Stress and resilience

## Abstract

Despite well-known peripheral immune activation in posttraumatic stress disorder (PTSD), there are no studies of brain immunologic regulation in individuals with PTSD. [^11^C]PBR28 Positron Emission Tomography brain imaging of the 18-kDa translocator protein (TSPO), a microglial biomarker, was conducted in 23 individuals with PTSD and 26 healthy individuals—with or without trauma exposure. Prefrontal-limbic TSPO availability in the PTSD group was negatively associated with PTSD symptom severity and was significantly lower than in controls. Higher C-reactive protein levels were also associated with lower prefrontal-limbic TSPO availability and PTSD severity. An independent postmortem study found no differential gene expression in 22 PTSD vs. 22 controls, but showed lower relative expression of *TSPO* and microglia-associated genes *TNFRSF14* and *TSPOAP1* in a female PTSD subgroup. These findings suggest that peripheral immune activation in PTSD is associated with deficient brain microglial activation, challenging prevailing hypotheses positing neuroimmune activation as central to stress-related pathophysiology.

## Abstract (web summary)

Neuroinflammation has been proposed to accompany the peripheral inflammation observed in PTSD. Here, authors find lower in vivo and postmortem levels of neuroimmune marker TSPO (translocator protein) in PTSD, in association with greater PTSD severity and higher plasma CRP.

## Introduction

Posttraumatic stress disorder (PTSD) develops in 6–10% of persons experiencing a traumatic event^1–3^. Existing interventions for PTSD have relatively low efficacy^4^; this has generated interest in identifying biomarkers, particularly immune markers, that may underlie the development and persistence of PTSD symptoms. C-reactive protein (CRP) is an acute phase reactant protein secreted by the liver in response to pro-inflammatory cytokine signaling. Higher levels of CRP have been linked with exposure to a traumatic event^5^, greater risk for developing PTSD following a traumatic event^6^, and greater severity of PTSD symptoms^6–8^. These findings, as well as its translational potential due to widespread use in clinical settings^9,10^, lend support for CRP as a critical target of investigation in relation to brain immunopathology in PTSD. More variability exists across studies of inflammatory cytokines in PTSD. Meta-analyses have associated traumatic experiences with higher levels of pro-inflammatory interleukin 6 (IL-6), interleukin 1β (IL-1β), and tumor necrosis factor α (TNF-α)^5^, and have found PTSD to be associated with higher levels of IL-6, IL-1β, and interferon γ (IFN-γ), but not TNF-α^11^. In contrast with these findings, subsequent studies have observed lower levels of TNF-α and IFN-γ immediately following trauma in individuals who later developed PTSD^12^, and higher levels of TNF-α in a combat-exposed PTSD group^13^. Investigations of cytokine levels in cerebrospinal fluid (CSF) have also been mixed. Some studies showed higher levels in PTSD of IL-6 and IL-1β compared to trauma-exposed^14^ and non-exposed individuals^15^, respectively, while others reported no differences in multiple pro-inflammatory cytokines between PTSD and non trauma-exposed individuals^16^. Despite these inconsistencies, more recent reviews have corroborated previous meta-analyses, providing additional evidence for elevation of pro-inflammatory IL-6, CRP, IFN-γ, and TNF-α in PTSD, using this as a basis to advocate for testing anti-inflammatory treatments^17,18^.

Peripheral immune modulators, including cytokines, are thought to affect PTSD pathophysiology by traversing the blood–brain barrier and acting on microglia, the resident immune cells of the central nervous system. These actions have been thought to trigger immunologic activation of microglia, which subsequently release pro- and anti-inflammatory cytokines that modify the brain response to stress, as observed in rodent models of stress and PTSD^19–21^. Many such preclinical models have observed “classical activation” or pro-inflammatory responses of microglia in stress-responsive regions of the brain, with increased stimulated cytokine levels or expression of IL-1β, IL-6, and TNF-α^19,20,22^ or increased microglia-specific proteins and cell-surface markers^22,23^. However, other rodent studies have demonstrated no changes or variable changes in pro-inflammatory cytokines^24,25^, or evidence of “alternately activated” microglial response with decreased pro- but increased anti-inflammatory cytokines^21,26^. The variability of neuroimmune responses to severe stress in these models may depend on differences in species, as well as duration, type, and intensity of stress paradigms, and behaviors assessed. The differing findings might also reflect true heterogeneity in stress-induced neuroimmune pathology, but these animal models are likely limited in their ability to recapitulate the complex pathophysiology and symptomatology that characterizes PTSD in humans.

The inconsistency of peripheral and neural immune alterations in association with PTSD pathophysiology underscores the importance of studying the neuroimmune system in the brains of humans living with PTSD. The goal of our study was to use the radiotracer [^11^C]PBR28 and positron emission tomography (PET) brain imaging to measure a marker of the neuroimmune system in PTSD. [^11^C]PBR28 targets the 18-kDa translocator protein (TSPO), which is a mitochondrial outer membrane protein expressed in microglia, and a molecule that has been extensively studied as a biomarker of microglia in humans with PET brain imaging^27^. [^11^C]PBR28 is a second-generation PET radioligand with high specific binding to TSPO^28^, favorable kinetics, and good reproducibility in quantifying TSPO availability^29^. Careful characterization by our group has demonstrated that [^11^C]PBR28 PET is sensitive to a decrease in microglial number after pharmacological depletion^30^ as well as LPS-induced increases in microglial activation in non-human primates^31^ and humans^32^.

The present study is the first to undertake an investigation of TSPO in vivo in individuals with PTSD, and to relate this neuroimmune system marker to PTSD symptom severity, peripheral immune markers, and transcriptomic alterations in postmortem brain tissue in PTSD. Based on the large literature demonstrating higher pro-inflammatory peripheral immune markers in PTSD^11^, we hypothesized that higher TSPO availability, measured by [^11^C]PBR28 PET, would be associated with greater PTSD symptom severity, and also with a diagnosis of PTSD. The primary examination of symptom severity reflects a deliberate dimensional approach to studying a disorder as heterogeneous in symptom presentation as PTSD. Our investigation was focused on a prefrontal-limbic circuit comprised of the amygdala, anterior cingulate cortex (ACC), hippocampus, insula, and ventromedial prefrontal cortex (vmPFC), which together are heavily implicated as part of neuroimmune^23,24,33–35^, neuroendocrine^36^ and transcriptomic^37^ networks in PTSD. Additionally, we sought to examine the relationship between peripheral immune markers and TSPO availability. Finally, in an independent dataset of postmortem brain tissue from individuals with and without PTSD, we examined transcript expression of *TSPO* and other microglia-associated genes that may be differentially expressed in relation to PTSD, to better understand what is likely a complex interaction of TSPO with other molecules to produce PTSD-related neuroimmune system alterations.

This study presents the first known evidence that, contrary to our hypothesis, lower prefrontal-limbic TSPO availability is significantly associated with greater PTSD symptom severity, and is significantly lower in individuals with a diagnosis of PTSD compared to controls. We confirm the association of peripheral inflammation, as measured by plasma CRP levels, with PTSD severity, and demonstrate that TSPO availability is negatively associated with CRP. In an independent sample of postmortem brain, we also report evidence of lower expression levels of *TSPO* and microglia-associated genes, *TNFRSF14* and *TSPOAP1*, in prefrontal cortical tissue from females with PTSD relative to non-PTSD controls. Collectively, these findings suggest that peripheral immune activation in PTSD is associated with suppression of microglia. Thus, these data question the presence of neuroimmunologic activation in PTSD, one of the prevailing hypotheses on the pathophysiology of PTSD in the literature, and suggest that medications to restore neuroimmune function may be effective for the treatment of PTSD.

## Results

Participant characteristics and injection parameters (Table 1) did not differ in the PTSD vs. control group with the exception of injected mass per body weight, which was significantly higher in the control compared to PTSD group. There was no significant effect in a linear regression analysis of injected mass per body weight on prefrontal-limbic TSPO availability across all participants (*p* = 0.22), so this was not adjusted for in subsequent modeling analyses. TSPO availability was higher in HABs vs. MABs, and data from the two excluded outliers were greater than 3 SD above the mean TSPO availability within HABs (Supplementary Fig. 1). Interpersonal trauma (sexual, physical, childhood) was more prevalent in the PTSD compared to control group.

**Table 1:** Participant characteristics and injection parameters.

Characteristics	Control (*n* = 26)	PTSD (*n* = 23)	*p*-value
Age: years	32 ± 12	38 ± 10	0.10
Sex	8 F, 18 M	10 F, 13 M	0.36
Body mass index	27.3 ± 4.3	27.8 ± 5.4	0.68
*rs6971* Genotype	17 HAB, 9 MAB	18 HAB, 5 MAB	0.32
Ethnicity (%)			0.32
African American	6 (23)	9 (39)	
Asian/Pacific Islander	1 (4)	1 (4)	
Caucasian	12 (46)	9 (39)	
Hispanic	7 (27)	4 (17)	
**Clinical Characteristics**			
PTSD severity			
CAPS-IV (*n*)	—	71 ± 24 (10)	—
CAPS-5 (*n*)	—	32 ± 10 (13)	—
Trauma exposure(s)	(*n *= 16)		
Sexual trauma	3	12	—
Physical trauma	10	21	—
Childhood trauma	11	23	—
Combat	3	9	—
Accident/natural disaster	13	17	—
Years since primary trauma (*n*)	16 ± 10 (12)	16 ± 11 (22)	0.91
Current comorbid MDD	—	18	—
Currently on psychotropic medication	—	5	—
Current tobacco users	12	8	0.41
Current cannabis users	4	7	0.21
Current alcohol users	17	13	0.53
**Injection parameters**			
Injected dose (MBq)	502.6 ± 189.3	573.9 ± 165.7	0.17
Injected mass (µg/kg)*	0.03 ± 0.03	0.01 ± 0.01	0.022
*f*_P_	0.024 ± 0.008	0.020 ± 0.007	0.14

*significant at *p* < 0.05; assessed from two*-*sided independent samples *t*-test.

Values are Mean ± SD, unless otherwise specified.

### Lower TSPO is associated with greater PTSD symptom severity

**Fig. 1:**
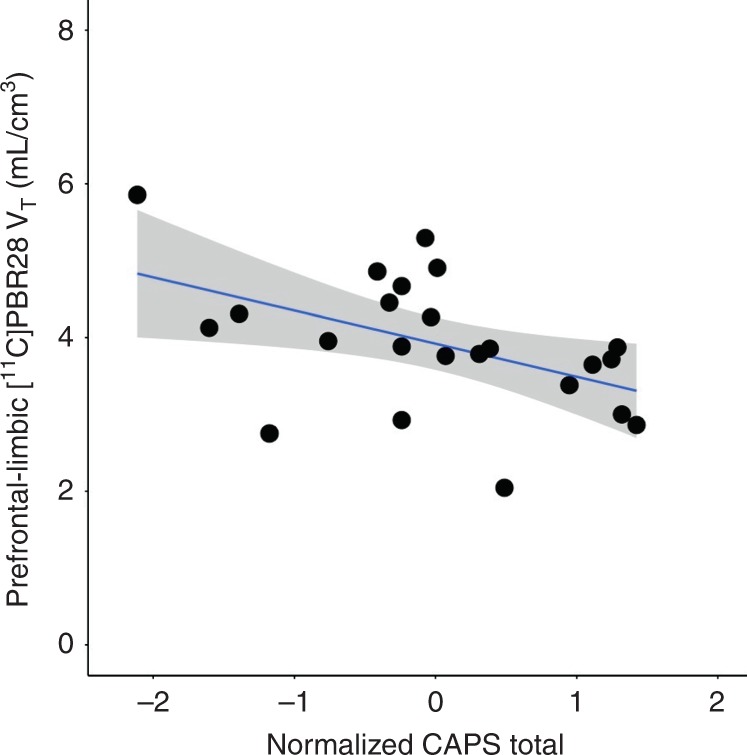
Prefrontal-limbic TSPO availability was associated with PTSD severity. Lower [^11^C]PBR28 *V*_T_ was significantly associated with greater PTSD severity (*R*^2^ = 0.51, *p* = 0.003; *β* = −0.43, *p* = 0.046), quantified by normalized total severity scores on CAPS in the PTSD group (*n* = 23). Coefficient of determination and standardized coefficients were assessed using linear regression. Displayed composite prefrontal-limbic [^11^C]PBR28 *V*_T_ values are genotype- and sex-adjusted, with gray shading indicating 95% confidence intervals.

In the PTSD group, lower composite prefrontal-limbic [^11^C]PBR28 distribution volume (*V*_T_) was significantly associated with greater severity of PTSD symptoms (Fig. 1**:** *β* = −0.43, *p* = 0.046) measured by total scores on the Clinician Administered PTSD Scale (CAPS). To investigate whether specific symptom clusters may be driving the association with overall symptom severity, we conducted post hoc analyses with PTSD symptom clusters, but observed no significant associations with CAPS re-experiencing, avoidance, numbing, and hyperarousal subscores. Sensitivity analyses revealed that the overall association of composite prefrontal-limbic TSPO availability with PTSD symptoms remained significant after adjusting for tobacco, cannabis, and alcohol use (all *β* > −0.43, all *p* < 0.05). Though there were trending effects (*p* < 0.10) of comorbid MDD and psychotropic medication use on overall PTSD symptom severity, there were no independent effects of comorbid MDD (*p* = 0.42) or psychotropic medication use (*p* = 0.20) on prefrontal-limbic TSPO availability.

### Group differences in TSPO availability

**Fig. 2:**
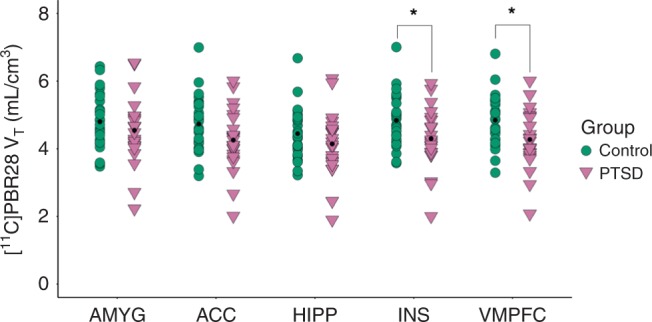
Prefrontal-limbic circuit TSPO availability was lower in the PTSD vs. control group. [^11^C]PBR28 *V*_T_ in the prefrontal-limbic circuit composed of amygdala, anterior cingulate cortex, hippocampus, insula, and vmPFC, was lower (*F*_5,41_ = 2.46, *p* = 0.049) in the PTSD (pink triangles, *n* = 23) compared to control (green circles, *n* = 26) group. A 14% difference in [^11^C]PBR28 *V*_T_ between groups was observed in insula (*p* = 0.036) and vmPFC (*p* = 0.023). Group differences were assessed using MANOVA and within-ROI comparisons assessed using ANOVA. Displayed [^11^C]PBR28 *V*_T_ values are adjusted for genotype and sex, with group-wise mean indicated by black point. AMYG: amygdala, ACC: anterior cingulate cortex, HIPP: hippocampus, INS: insula, VMPFC: ventromedial prefrontal cortex. *p < 0.05, not corrected for multiple comparisons.

Analysis in the primary regions of interest comprising a prefrontal-limbic circuit (i.e., amygdala, ACC, hippocampus, insula, vmPFC) revealed that [^11^C]PBR28 *V*_T_ was significantly lower in the PTSD than the control group (Fig. 2: MANOVA: *F*_5,41_ = 2.46, *p* = 0.049), reaching regional significance in the insula (14% lower, *p* = 0.036) and vmPFC (14% lower, *p* = 0.023).

Sensitivity analyses showed that the overall PTSD vs. control group difference in prefrontal-limbic TSPO availability remained significant after adjusting for tobacco, cannabis, and alcohol use (all *F* > 2.47, all *p* < 0.05). There were no differences in prefrontal-limbic TSPO availability between individuals in the PTSD group with or without comorbid MDD (*p* = 0.60) or current psychotropic medication use (*p* = 0.59), and between individuals in the control group with or without trauma exposure (*p* = 0.85).

A global pattern of lower [^11^C]PBR28 *V*_T_ in the PTSD group was visually apparent (Supplementary Fig. 2) with [^11^C]PBR28 *V*_T_ across all regions displayed in Supplementary Table 1, but a multivariate analysis showed no main effect of PTSD vs. control group across non-primary ROIs (MANOVA: *F*_10,36_ = 1.07, *p* = 0.41). Based on our finding of a significant relationship between TSPO availability and symptom severity, *post hoc* analyses were conducted among PTSD subgroups stratified by median of total symptom severity on the CAPS. In the high-severity PTSD compared to control group (but not in low-severity vs. control), [^11^C]PBR28 *V*_T_ in the prefrontal-limbic circuit was even lower (MANOVA: *F*_5,29_ = 3.34, *p* = 0.021) than in the overall PTSD vs. control group comparison, reaching regional significance in ACC (19% lower, *p* = 0.022), insula (21% lower, *p* = 0.009), and vmPFC (22% lower, *p* = 0.005) (Supplementary Fig. 3). There were no significant differences between high- vs. low-severity PTSD groups.

### C-reactive protein levels

**Fig. 3:**
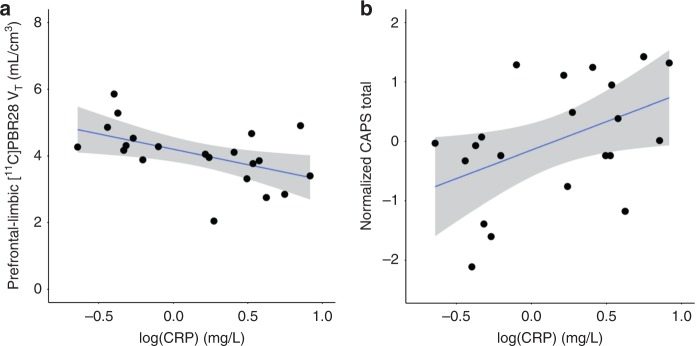
CRP was negatively associated with prefrontal-limbic TSPO availability and positively associated with PTSD severity. **a** Lower [^11^C]PBR28 *V*_T_ was significantly associated with higher levels of CRP (*R*^2^ = 0.52, *p* = 0.005; *β* = −0.92, *p* = 0.029) in the PTSD group (*n* = 21). Displayed [^11^C]PBR28 *V*_T_ values are adjusted for genotype and sex. **b** Higher CRP was significantly associated with greater PTSD severity (*R*^2^ = 0.21, *p* = 0.037) in the PTSD group (*n* = 21). Coefficient of determination and standardized coefficients were assessed using linear regression. Gray shading indicates 95% confidence intervals.

Driven by the evidence for CRP as a robust and differentially modulated peripheral immune marker in PTSD, blood CRP was assayed in a majority of participants (41 total—21 PTSD, 20 control). In the PTSD group, CRP levels were significantly negatively associated with composite prefrontal-limbic [^11^C]PBR28 *V*_T_ (Fig. 3a: *β* = −0.92, *p* = 0.029), and significantly positively associated with greater PTSD symptom severity measured as a normalized total CAPS score (Fig. 3b: *R*^2^ = 0.21, *p* = 0.037), confirming previous observations of an association between elevated CRP and PTSD symptomatology^7,8^. In the group as a whole, CRP levels were significantly negatively associated with composite prefrontal-limbic [^11^C]PBR28 *V*_T_ (*β* = −0.82, *p* = 0.003). This finding also held within the control group (*n* = 20, *β* = −0.76, *p* = 0.044). Both findings remained significant after removal of the single CRP sample analyzed by a different assay.

### Scan-day cytokine levels

Relationships between peripheral cytokine levels and TSPO availability were of interest, but these analyses were conducted in an exploratory manner, due to the lack of previously demonstrated associations between measured peripheral cytokines and TSPO availability, in either baseline or LPS-induced inflammatory states^32^. IFN-γ was associated with prefrontal-limbic [^11^C]PBR28 *V*_T_ (Supplementary Fig. 4: *β* = −0.64, *n* = 36, *p* = 0.046) across all participants, with an effect for interaction of group by IFN-γ level (*p* = 0.002) reflected in a negative association within the controls (*β* = −0.65, *n* = 20, *p* = 0.031) and a positive association within the PTSD group (*β* = 0.60, *n* = 16, *p* = 0.17) of IFN-γ with prefrontal-limbic [^11^C]PBR28 *V*_T_. However, these effects were not significant after correction for multiple comparisons. No other cytokine levels were significantly associated with prefrontal-limbic TSPO availability (Supplementary Table 2) or found to be significantly different in the PTSD compared to control group (Supplementary Table 3).

### Immune gene alterations in postmortem brain transcriptome

**Fig. 4:**
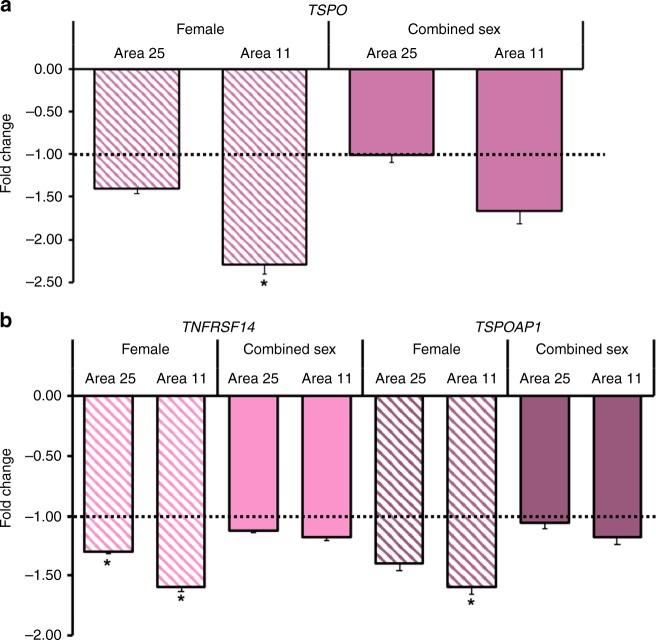
Expression of *TSPO*, *TNFRSF14*, and *TSPOAP1* genes was lower in areas comprising the prefrontal cortex in a postmortem brain sample. **a** In females with PTSD (*n* = 11) relative to female controls (*n* = 11), *TSPO* expression was significantly lower in BA11 (−2.30-fold lower (±0.10), *p* = 0.01), but not in BA25. Prefrontal cortex *TSPO* expression was not significantly lower in PTSD (*n* = 22) relative to controls (*n* = 22) combined across sex. **b** Relative expression of *TNFRSF14 was* significantly lower in BA11 (−1.60-fold (±0.03), *p* = 0.01) and in BA25 (−1.30-fold (±0.02), *p* = 0.04) in the female PTSD subgroup (*n* = 11 per subgroup). Relative expression of *TSPOAP1* was significantly lower in BA11 (−1.60-fold (±0.06), *p* = 0.04) in the female PTSD subgroup (*n* = 11 per subgroup). Apparent lower relative expression did not reach statistical significance for *TSPO* and *TSPOAP1* in BA25 in the female subgroup, and for *TSPO*, *TNFRSF14*, and *TSPOAP1* in BA11 in PTSD vs. controls combined across sex. Mann-Whitney U tests were used to assess differences in fold change. Displayed values are shown as fold change (−log_2_(dd*C*_T_))^-1^, representing the fold change in transcript expression, or the ratio of the average d*C*_T_ in the PTSD relative to average d*C*_T_ in the control group. Error bars indicate the log-corrected SD of d*C*_T_ calculated from the comparison PTSD sample. *p < 0.05, after Bonferroni correction for multiple comparisons for two exploratory genes.

Given both the large magnitude difference in TSPO availability that was observed in vmPFC, and the limited extant literature implicating BA25 and BA11 as regions of differential gene expression in PTSD^38,39^, postmortem analyses targeted the prefrontal cortical areas BA25 (subgenual prefrontal cortex) and BA11 (orbitofrontal cortex). *TSPO* expression was non-significantly lower in prefrontal cortex BA11 (−1.66-fold lower (±0.16), *p* = 0.07) and in BA25 (1.02-fold lower (±0.08), *p* = 0.33) in tissue from individuals with PTSD (*n* = 22) relative to non-PTSD controls (*n* = 22) (Fig. 4a). The possibility that effects of group might be obscured by not accounting for effect of sex on *TSPO* expression was investigated, based on evidence of sex effects on TSPO availability in the present and previous PET analyses^40^ and of sex effects on *TSPO* gene expression in the brain in preclinical studies^41^. There was a significant effect of sex (*p* = 0.048) on *TSPO* expression in BA11 in PTSD relative to controls, and a non-significant interaction effect (*p* = 0.32). Contrasting findings for *TSPO* expression in within-group, female vs. male comparisons also suggested an interaction. Gene expression was significantly higher in female relative to male controls (BA11: 2.60-fold higher (±0.10), *p* = 0.002; BA25: 2.30-fold higher (±0.08), *p* = 0.04) but lower, albeit non-significant, in females vs. males with PTSD (BA11: 1.4-fold lower (± 0.91), *p* = 0.50; BA25: 1.2-fold lower (± 0.70), *p* = 0.85). Thus, secondary analyses were conducted to investigate gene expression in a sex-specific manner in female and male subgroups. In females with PTSD (*n* = 11), *TSPO* expression was significantly lower relative to female controls (*n* = 11) in BA11 (−2.30-fold lower (±0.10), *p* = 0.01) but did not reach significance in BA25 (−1.40-fold lower (±0.06), *p* = 0.08) (Fig. 4a). There was no difference in relative *TSPO* expression in males with PTSD compared to male controls in either region. Exploratory analyses examined expression of microglia-associated genes, *TNFRSF14* and *TSPOAP1*, to provide a broader characterization of microglial regulation. There was evidence of non-significant group × sex interaction effects on differential expression of *TNFRSF14* (BA11: *p* = 0.063; BA25: *p* = 0.15) and *TSPOAP1* (BA11: *p* = 0.055; BA25: *p* = 0.12), resulting in subsequent sex-specific investigations of differential expression of these genes in PTSD vs. controls. *TNFRSF14* expression was significantly lower in prefrontal cortical areas in females with PTSD relative to female controls (BA11: −1.60-fold lower (±0.03), *p* = 0.01; BA25: −1.30-fold lower (±0.02), *p* = 0.04). In females with PTSD, there was also significantly lower relative expression of *TSPOAP1* in BA11 (−1.60-fold lower (±0.06), *p* = 0.04) but not in BA25 (−1.40-fold (±0.06), *p* = 0.05) (Fig. 4b).

There was no difference in males with PTSD compared to male controls in relative expression of these microglia-associated genes.

## Discussion

This study sheds new light on alterations in microglial regulation associated with PTSD based on data from in vivo PET imaging using [^11^C]PBR28 and analysis of microglia-associated gene expression changes in post-mortem brain tissue. The present results replicated the widely reported finding of higher blood CRP levels in relation with PTSD severity, suggestive of a pro-inflammatory state in PTSD. However, this study found significantly lower TSPO availability in prefrontal-limbic regions in PTSD. Both higher CRP levels and greater PTSD severity were associated with lower rather than higher TSPO availability. Finally, postmortem brain transcriptome analysis in an independent sample revealed lower expression of *TSPO, TSPOAP1* and *TNFRSF14* genes in prefrontal cortical tissue from females but not males with PTSD, providing convergent evidence of compromised microglial function potentially representing an overall neuroimmune suppression.

Compromised microglial function could contribute meaningfully to the pathophysiology of PTSD, particularly compromised cortico-limbic connectivity^42,43^. Microglia have been implicated in the immunologic regulation of synaptic plasticity in part through production of neurotrophic factors such as IGF-1 and BDNF^44,45^, and in a neuroprotective response in rodent models of spinal cord injury, with selective microglial depletion exacerbating neurodegeneration and motor impairment^45^. Adding to this is a growing consensus that higher TSPO levels do not simply represent neuroinflammatory M1-type microglial activation, but rather that TSPO likely represents a dynamically-regulated balance between microglial M1- and M2-type activation states. For instance, observations of M1-predominant activation did not result in any increase in TSPO in in vitro human microglia cell culture^46^, while TSPO overexpression was associated with M2-predominant activation and reduced pro-inflammatory cytokine production in rodent microglial cells^47^. Furthermore, direct viral overexpression of brain TSPO prior to a footshock stressor promoted a neuroprotective function and ameliorated the ensuing PTSD-like behaviors in rodents^34^. Thus, we surmise that lower TSPO availability in association with PTSD severity may reflect lower microglial TSPO expression related to reduced activation of a neuroprotective microglial type, or progressive neuroimmune suppression marked by depletion of neuroprotective microglia themselves, or possibly, the presence of both processes. TSPO PET would be sensitive to such alterations based on our group’s previous demonstrations that TSPO PET can detect increases in TSPO expression^31^ as well as microglial depletion^30^. While it is not clear whether low levels of TSPO are cause or consequence of PTSD, results of these studies indicate a suppressed ability to mount an adequate neuroprotective microglial response, which may in turn contribute to the chronicity and severity of PTSD. Longitudinal studies are required to disentangle possible causal and temporal relationships between trauma, neuroimmune responses, and PTSD symptoms.

Our investigation of peripheral immune markers in relation to the neuroimmune system revealed that lower TSPO was significantly associated with higher levels of the acute phase immune reactant, CRP, while confirming a significant association of higher CRP with greater PTSD severity in our PET study sample. Eraly et al. found that higher CRP prior to combat-related trauma was a stronger predictor for developing PTSD and hyperarousal and numbing symptoms^6^, and Michopoulos et al. found CRP levels to be positively associated with overall PTSD severity^7^. Thus, our data in combination with previous findings suggest that higher CRP may be a risk factor, while its association with lower prefrontal-limbic TSPO suggests a possible circuit-specific mechanism linking higher CRP with the development or maintenance of clinical PTSD pathologies. No significant associations were found in exploratory cytokine analyses, due to limited post hoc power after correction for the 7 different cytokines assayed. Overall, in contrast to previous suppositions of higher levels of all peripheral immune markers driving greater “neuroinflammation”, our finding of higher CRP in association with lower TSPO availability lends further support for a peripheral-to-neural immune system relationship that is more complex than previously proposed.

Results of our PET study converged with postmortem brain analyses showing lower relative gene expression—statistically significant only in females with PTSD—of prefrontal cortical *TSPO*; *TNFRSF14*, which encodes TNF-α receptors expressed on microglia; and *TSPOAP1*, encoding TSPO-associated protein 1, also known as peripheral benzodiazepine receptor-associated protein 1 (PRAX1). These data provide evidence of altered postmortem expression of *TSPO* and *TSPO-*related genes in humans with PTSD, adding to recent findings of reduced dorsolateral prefrontal cortex *IL1A* gene expression in PTSD^48^. The observation in PTSD of lower relative expression of *TNFRSF14* concurrently with *TSPO*, may reflect a reduction of microglia that are responsive to pro-inflammatiory TNF-α signaling and, in fact, parallels the association, though non-significant, of lower TSPO availability with lower peripheral TNF-α levels in our PET study. The additional finding of lower relative expression of *TSPOAP1* is of further interest, considering recent studies demonstrating upregulation of *Tspoap1* mRNA in hippocampus and amygdala of non-stressed rats in response to antidepressant treatment^49^. Lower expression of these three genes in PTSD may thus represent a downregulation of a network of genes associated with neuroprotective microglial function and, encouragingly, such gene network dysregulation may be modifiable^49^.

It is important to note the sex-specific nature of the postmortem findings. We explored possible effects of sex in both PET and postmortem studies, based on compelling evidence of sex differences in both brain TSPO availability from a recent multi-site study with [^11^C]PBR28 PET^40^, and of an apparent group by sex interaction effect on *TSPO* gene expression in our study, supported by evidence of differential effects of sex on *TSPO* expression in brain and other tissues in several preclinical studies^41,50^. Though we were not powered to conduct sex-specific subgroup analyses within the PTSD group in the PET study, we covaried for sex based on the improved statistical fit of our regression models of the PET data. In our postmortem study, we found that *TSPO* expression was significantly lower in a female PTSD relative to control subgroup, even though it was non-significantly lower in PTSD relative to controls combined across sex. This highlights the importance of sex-specific analysis of postmortem *TSPO* gene expression, and is underscored by the demonstration of sex differences in baseline *TSPO* gene expression^41^ and sex steroid-modulated regulation of *TSPO* expression and abundance of TSPO protein^50^, which may extend to a role for sex in the differential regulation of *TSPO* gene expression and TSPO availability in our present study as well. The findings nevertheless represent the first known corroboration of neuroimmune suppression in individuals with PTSD between an independent in vivo assessment of TSPO availability and ex vivo assay of *TSPO* gene transcription.

The divergence of our observation of lower TSPO availability in PTSD from higher TSPO availability in major depressive disorder (MDD)^33,51,52^ is noteworthy. Distinct neuroimmune phenotypes of PTSD and MDD are in line with the “hypocortisolemic” HPA-axis phenotype observed in PTSD that is notably the opposite of the “hypercortisolemic” phenotype of MDD^53,54^. An association of neuroimmune suppression with HPA-axis suppression in PTSD is supported by evidence of reduced glucocorticoid signaling and associated reduced microglial activation in mice exposed to chronic unpredictable stress^55^. Furthermore, we observed no independent association of comorbid MDD in the PTSD group with TSPO availability, implicating the observed association as PTSD-specific. Collectively, these findings implicate a neuroimmune-neuroendocrine basis for the distinct clinical presentations and prognoses observed between PTSD and MDD^56^.

Some limitations of our study should be noted. First, our study samples are representative of the heterogeneity in the PTSD population for factors such as use of tobacco and psychotropic medications, and we recognize that these factors could contribute to greater heterogeneity in the relationship between TSPO availability and PTSD symptoms. However, we did not observe any relationship of these factors with TSPO availability, and we consider it a strength of the study that our sample is more clinically generalizable. Second, our analysis of relationships between TSPO availability and cytokine levels was limited by a few factors. Single time-point measurements of plasma cytokines do not provide the most reliable indicator of peripheral immune state in each individual. Additionally, though we imputed values below assay detection limits according to recommended methods, this approach could introduce bias in the measurements. These factors led to the exploratory nature of this analysis. Third, due to exclusion criteria for eligibility to participate in a PET scan, our sample happens to be relatively healthy in terms of BMI and lack of metabolic and cardiovascular disease other than hypertension. These disorders may be driving the elevation of pro-inflammatory cytokines that have been observed in other PTSD samples, but not in ours, and it is possible that PTSD with high medical comorbidity may have a distinct neuroimmune phenotype. Fourth, our PET and postmortem samples inevitably differ on some clinical characteristics. For instance, the prevalence of cardiovascular and overdose-related deaths in the postmortem dataset is somewhat inherent to samples available in the postmortem PTSD brain bank, but may also contribute to different degrees of lower TSPO observed between the postmortem and PET data. Additionally, the absence of comorbid MDD in the postmortem dataset may limit the cross-validity of the lower TSPO findings. Nevertheless, the complementary findings of lower prefrontal cortical *TSPO* expression in the postmortem PTSD-only dataset and the lack of association of comorbid MDD with lower TSPO availability in the PET study, if anything, provide further support for lower TSPO being more specifically associated with PTSD symptomatology and less likely to be driven by comorbid MDD. Fifth, our targeted *TSPO* gene qPCR was performed in subgenual prefrontal cortex (BA25) and orbitofrontal cortex (BA11) as an attempt to capture tissue comprising the greatest amount of the larger prefrontal cortex, but these regions are not precisely analogous to the AAL atlas-defined vmPFC region in which we found the greatest magnitude difference in TSPO availability in our PET study. Sixth, despite the extensive array of peripheral immune markers examined in association with TSPO availability, these analyses are not able to directly inform underlying mechanisms of microglial function in PTSD, such as delineation of the balance between M1 or “classical activation” representing neuroinflammation and M2 or “alternative activation” representing neuroprotection. Nevertheless, we assert that our associations of lower TSPO levels with greater symptom severity, peripheral immune markers, and alterations in other putatively neuroprotective microglia-associated genes, represent initial descriptive evidence of a functional microglial characterization which should, in the future, be incorporated into mechanistic models of microglial function specifically in the context of PTSD.

Taken together, with data from previous literature, our findings challenge previous conceptions of concurrent peripheral and neural inflammation in PTSD, instead providing support for a novel model of neuroimmune pathophysiology underlying PTSD: deficient neuroimmune neuroprotective function. We propose that this deficient neuroimmune neuroprotective function is characterized by lower TSPO and alterations in other microglia-associated molecular markers, *TNFRSF14* and *TSPOAP1*. The association of lower TSPO with greater PTSD severity, highlights the specificity of the observed neuroimmune dysregulation to PTSD clinical pathology. Additional associations of both lower TSPO and greater symptom severity with higher levels of CRP, previously shown to be a risk factor for PTSD, implicate higher CRP as a possible risk factor making individuals more vulnerable to developing this particular neuroimmune phenotype of PTSD. Importantly, preclinical studies provide hope that a chronic neuroimmune suppression phenotype may not be permanent. Ongoing experiments include assessment of the dynamic functioning of the neuroimmune system in PTSD to see if microglia mount a comparable response to the previously validated LPS challenge^32^, and to examine if LPS might even improve symptoms in a neuroimmune suppressed PTSD phenotype, similar to the LPS-induced reversal of numbing-like behaviors and accompanying microglial suppression observed in rodent PTSD models^35^. Direct agonism of TSPO may provide an even more specific manipulation of microglial activity, with overexpression of TSPO previously shown to reduce anxiety-like behaviors analogous to PTSD re-experiencing and hyperarousal symptoms^34^. Such avenues of investigation will help inform the development of pharmacological agents targeting microglia for potential therapeutic intervention to restore neuroimmune system function in a subset of individuals with a particularly severe and unremitting presentation of PTSD.

## Methods

### Participant recruitment

Forty-nine individuals participated in this study and were recruited from 2011 to present via advertisements in public forums or through clinician referral. Written informed consent was obtained from all participants after a complete explanation of study procedures. A Structured Clinical Interview for the DSM (IV or 5) was administered to all participants to screen for history of trauma, PTSD among trauma-exposed individuals, presence of comorbid Major Depressive Disorder in the PTSD group, and exclusion of all other psychiatric diagnoses, including alcohol dependence (DSM-IV) and alcohol use disorder (DSM-5) in both PTSD and control groups. Clinical characterization of PTSD (*n* = 23) was performed using the Clinician Administered PTSD Scale (DSM-IV: *n* = 10; DSM-5: *n* = 13) administered by trained clinical research assistants under the supervision of a licensed clinical psychologist. Social drinking, and tobacco and cannabis use were permitted and quantified using the past month Timeline Followback^57^. Current use of psychotropic medications, other than antipsychotics and anticonvulsant mood stabilizers, was permitted in the PTSD group, and was determined at screening and confirmed on scan-day (Total: *n* = 5 of 23, serotonin reuptake inhibitors: *n* = 3, norepinephrine-dopamine reuptake inhibitors: *n* = 1, GABA_A_ receptor agonists: *n* = 3, see Supplementary Table 4 for greater detail). Medical examination was performed and clinical laboratory tests were reviewed by the study physicians to confirm absence of significant medical illness- in particular individuals using non-steroidal and other anti-inflammatory medications on a daily or regular basis were excluded. All participants were screened for TSPO *rs6971* genotype (NeoGenomics Laboratories, Houston, TX, USA) and individuals with homozygous T/T at the *rs6971* locus or low-affinity binders were excluded, based on previous work establishing the effect of TSPO *rs6971* genotype on affinity of [^11^C]PBR28 for TSPO^58^. All screening and investigation procedures were in accordance with Code of Federal Regulations Title 45, Part 46 policy on protection of human subjects in research, and were approved and overseen by the Yale University Institutional Human Investigation Committee and the Yale New Haven Hospital Radiation Safety Committee.

### Trauma history assessment

History of at least one traumatic event was assessed in all participants by screening with the SCID Post Traumatic Stress Disorder (DSM-IV-*TR*) or SCID Trauma and Stress-related Disorders screen (DSM-5). History of particular types of trauma as listed in Table 1 was assessed by combination of participant report during clinician interview, self-report on the Life Events Checklist, a well-validated 17-item tool for assessing trauma history^59^, and the Childhood Trauma Questionnaire Short From (CTQ), a well-validated 28-item measure for assessing history of traumatic events before 18 years of age^60^.

### Imaging acquisition

Synthesis of [^11^C]PBR28 was achieved at high molar activity of 233 ± 173 MBq/nmol according to previously established methods^61^ and described in Supplementary Methods. Participants received a 1-min intravenous bolus of 536 ± 180 MBq [^11^C]PBR28 and were subsequently scanned for 120 min (apart from one scan terminated at 90 min due to participant discomfort) on the High-Resolution Research Tomograph (HRRT; Siemens, Medical Solutions, Knoxville, TN, USA). A T1-weighted structural MRI image acquired with a sagittal gradient-echo (MPRAGE) sequence (Siemens 3.0 T Prisma Fit; 176 sagittal slices, thickness = 1 mm, TR = 2530 ms, TE = 2.26 ms, flip angle = 7°, FoV = 256 mm, matrix size = 256 × 256, 1 × 1 × 1 mm^3^ voxels) was also obtained in all individuals for co-registration of PET images and for definition of anatomical regions of interest.

### C-reactive protein analysis

Levels of C-reactive protein were measured in 42 individuals. CRP level was measured on scan day in 41 participants using hs-CRP Wide Range Reagent (Cliniqa Corporation, San Marcos, CA, USA), and on initial screening day in 1 control participant using ultrasensitive CRP (Graham Massey Analytical Labs, Shelton, CT, USA). CRP levels greater than 10 were excluded from subsequent analyses.

### Cytokine analysis

Levels of cytokines IL-1β, IL-6, IL-8, IL-10, TNF-α, IFN-γ, and MCP-1 were measured in plasma prior to radiotracer injection in 37 individuals using the MILLIPLEX panel assay (MillporeSigma, Burlington, MA, USA). Measurements below the assay-specific detection limit were imputed as the assay-specific detection limit normalized by a factor of 2 or √2, previously shown to be appropriate for normal and log-normal distributions, respectively, of data with values below the detection limit^62^.

### Arterial input function measurement

An arterial catheter was placed in the radial artery contralateral to [^11^C]PBR28 injection site for arterial sampling throughout the duration of the scan. Blood samples were acquired to measure plasma radioactivity concentrations either manually every 10 s (*n* = 8) or continuously for the first 3 min using an automated blood sampler (PBS-101, Veenstra Instruments, Joure, Netherlands), and then at 3, 5, 8, 12, 15, 20, 25, 30, 40, 50, 60, 75, 90, 105, and 120 min in manual samples analyzed with a cross-calibrated well counter (1480 Wizard, Perkin-Elmer, Waltham, MA, USA), with merging of plasma radioactivity concentrations measured by automated and manual sampling. Fraction of un-metabolized [^11^C]PBR28 was measured using high-performance liquid chromatography (HPLC) as previously described^63^ in plasma samples taken at 3, 15, 30, 60, and 90 min. The metabolite-corrected arterial input function was calculated as the product of the plasma radioactivity and un-metabolized [^11^C]PBR28 fraction in plasma. Free fraction in plasma (*f*_P_) was measured in triplicate with ultrafiltration (Millipore Centrifree micropartition device 4104, Billerica, MA, USA) according to manufacturer guidelines with 4 mL of pre-injection arterial blood, to confirm no differences between groups. Values of *f*_P_ were calculated as the ratio of radioactivity in ultrafiltrate to total radioactivity in plasma.

### Imaging processing and analysis

PET data were acquired in list-mode and time-binned as follows: 6 × 0.5-min, 3 × 1-min, 2 × 2-min, and 22 × 5-min. Data were then reconstructed using the MOLAR algorithm^64^ with correction for participant head motion (Polaris Vicra Optical Tracking System, NDI Systems, Waterloo, Canada), attenuation, scatter, randoms, and dead time.

The following automatic anatomical labeling (AAL)-defined regions of interest (ROIs)^65^ were defined in template MRI space (Montreal Neurological Institute) and transformed first onto each individual participant’s MRI and then onto a 0–10 min summed PET image: amygdala, anterior cingulate cortex (ACC), caudate, cerebellum, frontal cortex, hippocampus, insula, occipital cortex, orbitofrontal cortex, parietal cortex, putamen, temporal cortex, thalamus, and ventromedial prefrontal cortex (vmPFC).

The Multilinear Analysis method (MA1 with t* = 30 min) with metabolite-corrected arterial input function was used to estimate regional [^11^C]PBR28 volume of distribution or *V*_T_^29^, defined as the equilibrium ratio of tissue to plasma radioactivity^66^.

### Postmortem gene expression analysis

Quantitative real-time PCR (qRT-PCR) was performed on dissected tissue from 22 donors with PTSD (mean age: 46 ± 11 years, 11 females) and 22 matched non-PTSD control donors (mean age: 48 ± 12 years, 11 females) shown in Supplementary Table 5, in two areas within the prefrontal cortex: the subgenual prefrontal cortex (sgPFC; BA25) and the orbitofrontal cortex (OFC; BA11). Clinical diagnosis was based on psychiatric history and demographic data information obtained by psychological autopsy performed postmortem. Trained clinicians with informants (usually next of kin) acquainted with the subject generated all of the necessary information for diagnosis. To avoid systematic biases, PTSD and control cases were characterized by the same psychological methods, and were evaluated for presence of PTSD as well as lifetime alcohol and substance abuse and dependence, and for exclusion of other psychiatric and neurological illness, as determined by interview screening, medical examiner documentation, and toxicology screening. Psychiatric narratives were determined by consensus diagnosis between two clinicians using DSM-IV Axis I lifetime diagnoses criteria^67^ using SCID-I interviews that were adapted for psychological autopsy^68^ and review of relevant medical records. At the time of death 15 of 22 donors with PTSD were on an antidepressant and 13 of 22 control donors were on an over the counter medication. Details of cause of death are in Supplementary Tables 6 and 7. The average postmortem interval (PMI) was 16.1 h in the PTSD cohort and 18.8 h for the controls. There were no significant differences between the PTSD and control samples in age, PMI, pH, or RNA integrity number.

qRT-PCR was performed using primers designed to detect transcripts of interest. mRNA was isolated from the sgPFC and OFC using the RNEasy Plus Mini Kit (Qiagen, Venlo, Netherlands); 1 µg of mRNA was reverse-transcribed into cDNA using the iScript cDNA Synthesis kit (Bio-Rad, Hercules, CA). RNA was hydrolyzed and re-suspended in nuclease free water. Gene specific primers for *TSPO* (F: ACAGAGAAGGCTGTGGTTCC, R: AGCAGGAGATCCACCAAGG)*, TSPOAP1* (F: CTTGGAGTTGTGTCGGAAGG; R: AGCAATCTGCTTGTCCTTGG), and *TNFRSF14* (F: CTGCAAGGAGGACGAGTACC, R: ATTGAGGTGGGCAATGTAGG) and the control housekeeping gene *GAPDH* (ACCCAGAAGACTGTGGATGG; GAGGCAGGGATGATGTTCTG) were designed using Primer 3 v.0.4.0 freeware (http://bioinfo.ut.ee/primer3-0.4.0/) and tested for efficiency and specificity by serial dilution and melt curve analysis. Sybr Green mix (Bio-Rad, Hercules, CA) was used to amplify cDNA. Fold regulation was calculated by using the 2–delta delta Ct (2-DDCt) method. The 2-DDCt analysis uses the threshold cycles (*C*_T_), calculated from the increasing fluorescent signal of the amplicon during PCR, to calculate relative fold change in expression of genes of interest between two samples (i.e., PTSD vs. control), with gene of interest normalized to a housekeeping gene as an internal control within each sample.

### Statistical analysis

Demographic and clinical characteristics, *rs6971* genotype, injection parameters, and peripheral inflammatory markers were compared across groups using Student’s *t*-tests for continuous variables and chi-squared tests for categorical variables. Distributions of all dependent variables were examined for normality using Shapiro-Wilk tests and inspection of histograms and Q-Q plots, and [^11^C]PBR28 *V*_T_ values exceeding mean ± 3 SD within *rs6971* genotype groupings were pre-defined as outliers. Data from two individuals (1 control, 1 PTSD) were accordingly excluded due to regional [^11^C]PBR28 *V*_T_ values that were 3.9 ± 0.6 SD above the mean within the HAB group, after confirming absence of technical issues in imaging and input function measurement, and absence of outlying *f*_P_ values, that may have contributed to outlying *V*_T_ values. This resulted in normal distributions of [^11^C]PBR28 *V*_T_ values in all ROIs, including a composite prefrontal-limbic ROI calculated as an average across the a priori ROIs (amygdala, ACC, hippocampus, insula, and vmPFC). Non-normal distributions of other dependent variables were examined for outliers and log-transformed as necessary. Sex, BMI, and age were examined as these factors were recently found to be significant predictors of TSPO availability in healthy individuals in a multi-site PET study^40^. Sex was the only factor found to improve statistical fit, by virtue of the Akaike Information Criterion, in the present analysis of TSPO availability in association with PTSD symptoms, and was therefore included in subsequent models.

A general linear modeling approach was used to examine the relationship between PTSD severity and TSPO availability in a composite prefrontal-limbic circuit, computed as an average across the a priori brain regions. For this and all subsequent linear modeling analyses, results were reported as *R*^2^, the coefficient of determination, and *β*, the standardized coefficient of regression for the predictor of interest in models with multiple predictors. Composite prefrontal-limbic [^11^C]PBR28 *V*_T_ was modeled as a dependent variable, with CAPS total and CAPS subscores as predictors, adjusting for *rs6971* genotype and sex. PTSD severity was quantified by CAPS total scores normalized separately for DSM versions IV and 5 within the PTSD group and then combined into a normalized variable for subsequent analyses. To explore possible associations between specific PTSD symptom clusters and TSPO, normalized CAPS subscores for re-experiencing, avoidance and hyperarousal were derived from the common items within respective criteria on the CAPS-IV and CAPS-5. CAPS numbing subscore was constructed from the items belonging to the numbing factor of the 5-factor model under DSM-IV^69^ and items belonging to the anhedonia factor in the 7-factor model under DSM-5^70^, which shares greatest overlap with the 5-factor DSM-IV model.

Analysis of differences in TSPO availability between the PTSD and control group was conducted using multivariate ANOVA with [^11^C]PBR28 *V*_T_ in a prefrontal-limbic circuit including ACC, vmPFC, amygdala, hippocampus, and insula entered as dependent variables, and with PTSD vs. control group, *rs6971* genotype and sex entered as fixed factors, with an overall significance threshold of *α* = 0.05. *Post hoc* analyses of high- vs. low-severity PTSD vs. control group were also conducted using the same multivariate ANOVA approach, whereby the PTSD group was stratified based on median total CAPS score (70 on CAPS-IV and 32 on CAPS-5).

Additional sensitivity analyses examined the possible effects on the relationship of TSPO availability and PTSD for variables including tobacco, cannabis and alcohol use, quantified using the past month Timeline Follow-back, comorbid MDD and psychotropic medication in the PTSD group, and trauma exposure vs. no exposure in the control group.

The relationship of log-transformed CRP and prefrontal-limbic TSPO availability was assessed using a linear modeling approach, adjusting for *rs6971* genotype and sex, and a general linear model was also used to compare log-transformed CRP and PTSD severity as measured by normalized CAPS total.

Exploratory analyses were conducted using linear modeling to assess possible relationships of prefrontal-limbic TSPO availability with levels of pro- and anti-inflammatory cytokines, and using two-sided Student’s t-tests to assess group differences in cytokine levels. The exploratory nature of these analyses was due to the lack of detection of reliable associations between baseline and LPS-stimulated cytokine levels with TSPO availability in a previous study^32^ as well as the need to impute values below the assay-specific detection limits. A Benjamini-Hochberg procedure with *α* = 0.05 was used for correction of multiple comparisons in *post hoc* and exploratory analyses. Uncorrected *p* values were reported for data not surviving false detection rate or FDR correction, and adjusted *p* values were reported for data surviving FDR correction. Analyses of imaging, symptom severity, and peripheral immune marker data were conducted in RStudio version 1.0.153 with R programming language version 3.4.1.

For the postmortem gene expression analysis, relative differences in transcript expression between PTSD and non-PTSD control donors were evaluated using Graphpad Prism v7 (Graphpad Software, San Diego, CA) and reported as fold changes calculated using the 2-DDCt method. Secondary sex-specific analyses of postmortem transcript expression were conducted, based on preclinical literature suggesting sex-specific regulation of *TSPO* gene expression throughout tissues including in corticolimbic tissue^41,50^. Mann Whitney U tests were used to assess statistical differences in the calculated fold regulation, followed by Bonferroni correction for multiple comparisons across the two exploratory genes of interest: *TNFRSF14* and *TSPOAP1*. Bonferroni-corrected *p* values were reported for data surviving correction for multiple comparisons.

### Reporting summary

Further information on research design is available in the Nature Research Reporting Summary linked to this article.

## Supplementary information

Supplementary Information

Reporting Summary

## Data Availability

Due to the sensitive nature of human participant information, data are available upon reasonable request by contacting the corresponding author. Minimal, de-identified source data for Figs. 1–3a, b, and 4a, b and Supplementary Figs. 1–3a, b, and 4a, b are available as a Source Data file.

## Source Data

Source Data

## Code availability

Software codes used for image pre-processing and analysis are custom and proprietary, but specific parts of code may be made available upon reasonable request.
