# Supplementary material for: PTSD is associated with neuroimmune suppression: evidence from PET imaging and postmortem transcriptomic studies
Source: Nat Commun. 2020 May 12;11:2360. doi: 10.1038/s41467-020-15930-5 (PMC7217830; doi:10.1038/s41467-020-15930-5)
Supplement: Supplementary file 1 — Supplementary Information [file 41467_2020_15930_MOESM1_ESM.pdf]

Supplementary Information

PTSD is associated with neuroimmune suppression: Evidence from PET imaging and postmortem transcriptomic studies

Bhatt et al.

Supplementary Figure 1: [^{11}C]PBR28 V_T across non-primary regions in PTSD and control groups. A pattern of lower [^{11}C]PBR28 V_T in PTSD was apparent but not statistically significant (*n* = 23 PTSD, *n* = 26 control: *p* = 0.41) across remaining brain regions. Group differences were assessed using MANOVA. Displayed [^{11}C]PBR28 V_T values are adjusted for genotype and sex, with group-wise mean indicated by black point. CAU: caudate, PUT: putamen, FC: frontal cortex, OFC: orbitofrontal cortex, OC: occipital cortex, PC: parietal cortex, TC: temporal cortex, CER: cerebellum, THA: thalamus

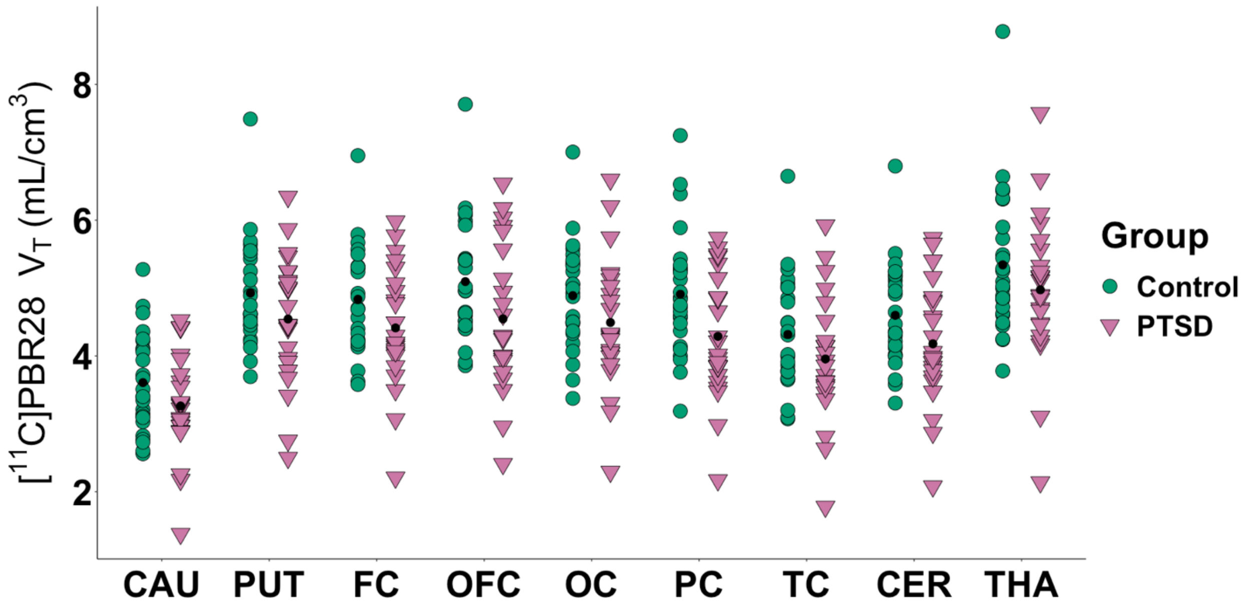

Supplementary Table 1: [^{11}C]PBR28 V_T across regions in PTSD and Control.

Region	Control		PTSD	
	**HAB (*n* = 17)**	**MAB (*n* = 9)**	**HAB (*n* = 18)**	**MAB (*n* = 5)**
**Amygdala***	5.1 ± 0.9	2.6 ± 0.6	4.6 ± 1.1	3.1 ± 1.1
**Anterior Cingulum***	5.0 ± 0.9	2.5 ± 0.6	4.3 ± 1.0	2.9 ± 0.9
**Hippocampus***	4.7 ± 0.8	2.5 ± 0.6	4.2 ± 1.0	3.0 ± 1.0
**Insula***	5.1 ± 0.8	2.5 ± 0.5	4.3 ± 1.0	2.8 ± 0.8
**Ventromedial PFC***	5.1 ± 0.8	2.6 ± 0.5	4.3 ± 0.9	2.9 ± 0.8
**Caudate**	3.9 ± 0.8	2.2 ± 0.7	3.4 ± 0.8	2.6 ± 0.8
**Putamen**	4.7 ± 0.9	2.4 ± 0.6	4.0 ± 0.9	3.0 ± 1.2
**Frontal Cortex**	5.1 ± 0.8	2.8 ± 0.6	4.5 ± 0.9	3.1 ± 0.9
**Orbitofrontal Cortex**	5.2 ± 0.8	2.8 ± 0.5	4.6 ± 1.1	3.1 ± 0.9
**Occipital Cortex**	5.3 ± 0.9	3.1 ± 0.6	4.6 ± 1.1	3.3 ± 1.1
**Parietal Cortex**	5.2 ± 1.0	2.8 ± 0.5	4.4 ± 1.0	3.1 ± 0.9
**Temporal Cortex**	4.9 ± 0.8	2.7 ± 0.6	4.3 ± 1.0	3.0 ± 0.9
**Cerebellum**	5.2 ± 0.8	2.7 ± 0.6	4.6 ± 1.0	3.1 ± 1.0
**Thalamus**	5.6 ± 1.1	2.7 ± 0.8	5.0 ± 1.2	3.1 ± 0.9
**Whole Brain**	4.8 ± 0.8	2.6 ± 0.4	4.2 ± 0.9	2.8 ± 0.7
**Composite**	5.0 ± 0.8	2.5 ± 0.6	4.4 ± 1.0	2.9 ± 0.9

**a priori regions of interest used to compute Composite* V_T

Values of [^{11}C]PBR28 V_T reported as mean ± SD for high affinity (HAB) and medium affinity binders (MAB) within control and PTSD groups.

Supplementary Figure 2: Prefrontal-limbic TSPO availability in PTSD group stratified by median PTSD severity into high- (filled triangles) and low-severity (empty triangles) of PTSD reveals step-wise relationship between lower TSPO and severity of PTSD most apparent in ACC, insula, and vmPFC. Prefrontal-limbic TSPO availability was significantly lower ($F_{5,29}$ = 3.34, p = 0.021) in the PTSD subgroup with high severity (n = 12) compared to control (n = 26): 19% lower in ACC (p = 0.022), 21% lower in insula (p = 0.009), and 22% lower in vmPFC (p = 0.005). Group differences were assessed using MANOVA and within-ROI comparisons assessed using ANOVA. Displayed [^{11}C]PBR28 V_T values are adjusted for genotype and sex, with group-wise mean indicated by black point. **p < 0.01, *p < 0.05

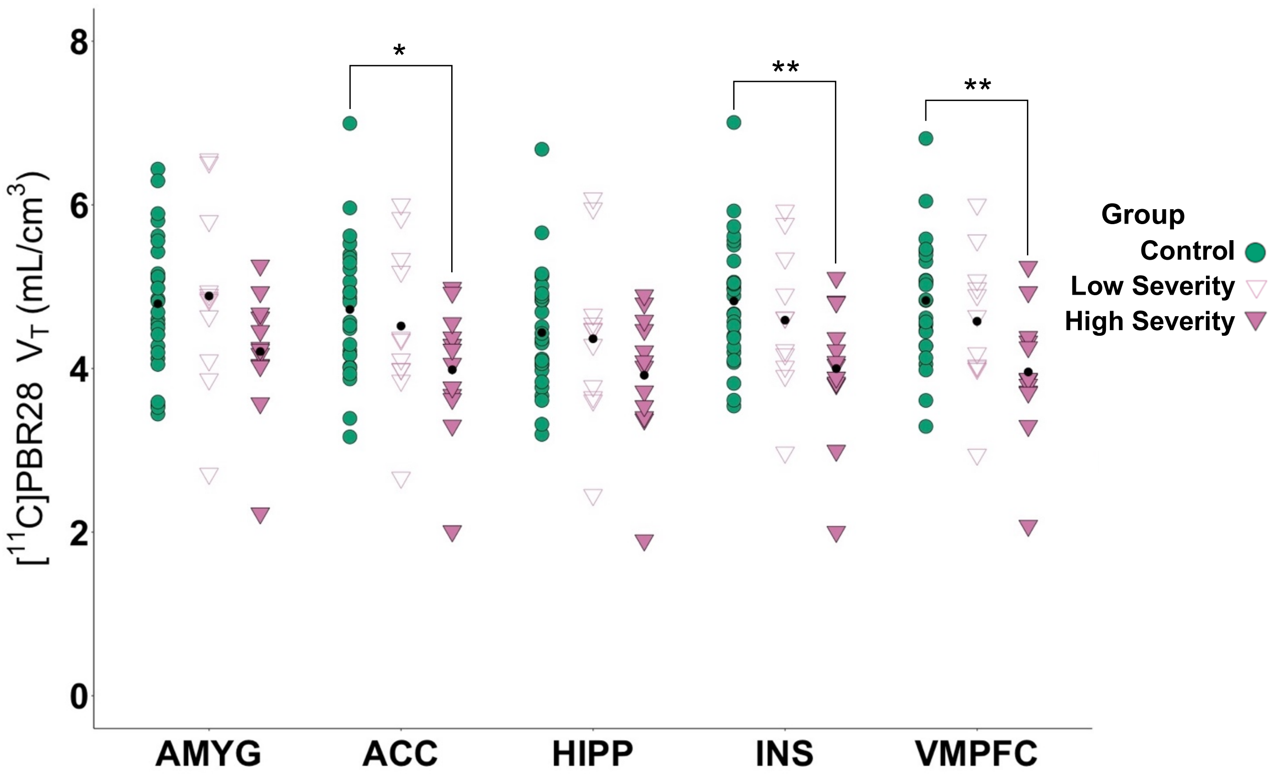

Supplementary Figure 3: TSPO availability distributed across HAB and MAB status determined by rs6971 genotype. **a** Individuals with MAB status (*n* = 5 PTSD, *n* = 9 controls) have lower [^{11}C]PBR28 V_T than individuals with HAB status (*n* = 18 PTSD, *n* = 17 controls) regardless of PTSD vs. control group status. **b** [^{11}C]PBR28 V_T values from excluded outliers (solid black) are shown, consistent with being outside of 3 SD from the mean within the HAB subgroup (black outline). PTSD (*n* = 24) vs. control group (*n* = 27) showed a trend-level difference (p = 0.062) when outliers were not excluded. Group differences were assessed using MANOVA. Displayed are [^{11}C]PBR28 V_T values with subgroup-wise mean indicated by black point. ^p < 0.10

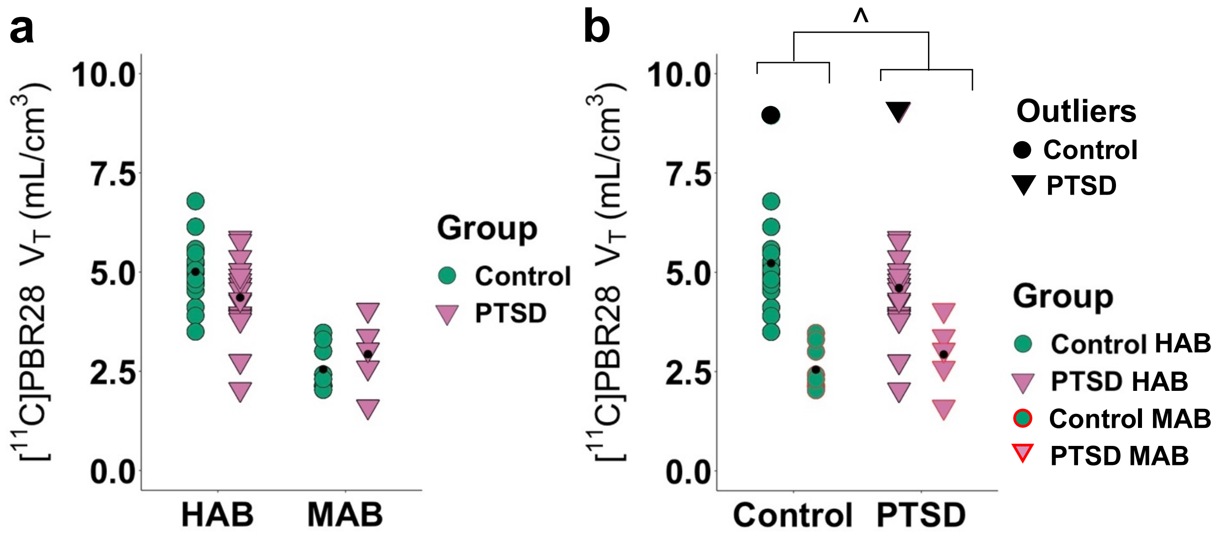

Supplementary Figure 4: Pro-inflammatory cytokine IFN-γ was positively associated with prefrontal-limbic TSPO availability. **a.** A positive directional, but non-significant, association between [^{11}C]PBR28 V_T and IFN-γ is apparent in the PTSD group (n = 16, p = 0.17). Displayed [^{11}C]PBR28 V_T values are adjusted for genotype and sex. **b.** Within controls (n = 20), [^{11}C]PBR28 V_T was negatively associated with IFN-γ (R^2 = 0.77, p < 0.001; β = -0.65, p = 0.031), though this association was not significant after FDR correction. Coefficient of multiple correlations and standardized coefficients were assessed from linear regressions. Displayed [^{11}C]PBR28 V_T values are adjusted for genotype, with gray shading indicating 95% confidence intervals.

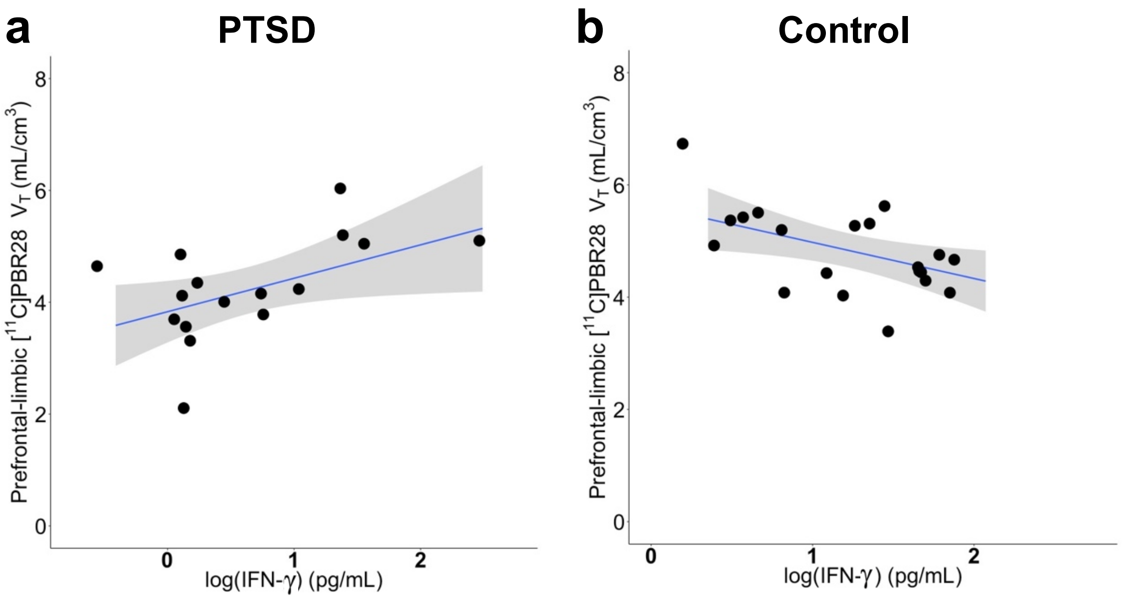

Supplementary Table 2: Exploratory regression analysis of cytokine levels in relation to prefrontal-limbic TSPO availability.

Cytokines	All		Control		PTSD	
	***β* (*n*)**	***p***	***β* (*n*)**	***p***	***β* (*n*)**	***p***
**Pro-inflammatory**						
Interleukin-1β	2.16 (29)	0.067	-1.90 (14)	0.097	2.49 (15)	0.058
Interleukin-6	0.11 (36)	0.59	-0.23 (19)	0.35	0.17 (17)	0.63
Tumor necrosis factor-α	-1.60 (35)	0.16	-1.73 (19)	0.095	1.43 (16)	0.18
Interferon-γ^	-0.64 (36)	0.046	-0.65 (20)	0.031	0.60 (16)	0.17
Monocyte chemotactic protein-1	0.93 (37)	0.13	1.01 (20)	0.18	0.51 (17)	0.60
Interleukin-8	-0.46 (37)	0.22	-0.39 (20)	0.27	0.32 (17)	0.45
**Anti-inflammatory**						
Interleukin-10	-0.38 (34)	0.48	-0.87 (17)	0.18	0.15 (17)	0.76

^*$p < 0.05$, not surviving FDR correction for multiple comparisons*

Standardized coefficients were assessed using linear regression.

Supplementary Table 3: Cytokine levels in PTSD vs. control group.

Cytokines	Control Mean ± SD (*n*)	PTSD Mean ± SD (*n*)	*p*-value
**Pro-inflammatory**			
Interleukin-1β	1.3 ± 0.6 (14)	1.2 ± 0.6 (15)	0.67
Interleukin-6	14.9 ± 27.6 (19)	9.4 ± 19.5 (17)	0.49
Tumor necrosis factor-α	10.6 ± 4.5 (19)	9.0 ± 5.3 (16)	0.35
Interferon-γ	33.3 ± 33.6 (20)	27.4 ± 76.0 (16)	0.74
Monocyte chemotactic protein-1	432.3 ± 222.9 (20)	426.6 ± 242.2 (17)	0.94
Interleukin-8	32.0 ± 43.0 (20)	12.7 ± 20.7 (17)	0.086
**Anti-inflammatory**			
Interleukin-10	7.2 ± 8.8 (17)	6.2 ± 7.0 (17)	0.68

P-values are derived from two-sided independent samples t-tests.

Supplementary Table 4: Details of psychotropic medication use.

Subject	Medication- dose/frequency
1	Prozac- 60 mg daily<br>Trazodone- 150 mg nightly<br>Klonopin- 0.5 mg nightly (did not take for 2 nights before the scan)
2	Citalopram- dose not available, daily<br>Trazodone- 25 mg prn
3	Ambien- 2.5 mg nightly
4	Pristiq- 100 mg daily<br>Trazodone- 100 mg nightly<br>Klonopin - 1 mg twice daily
5	Bupropion- 350 mg daily

Supplementary Table 5: Demographics Summary of Postmortem Tissue.

Variable	Control (*n* = 22)	PTSD (*n* = 22)		
	**Mean ± SD**	**Mean ± SD**	***t***	***p*-value**
Age at Death (years)	48 ± 12	46 ± 11	0.5	0.6
Sex (% female)	50	50	-	-
Race (% Caucasian)	77	82	-	-
PMI	18.8 ± 6.8	16.1 ± 5.6	1.6	0.2
pH	6.5 ± 0.3	6.5 ± 0.4	0.5	0.7
RIN	7.5 ± 1.0	7.6 ± 1.2	0.4	0.7

Values are mean ± SD unless otherwise specified. *P*-values are derived from two-sided independent samples t-tests. PMI: Postmortem interval, RIN: RNA integrity number

Supplementary Table 6: Comorbid diagnoses, manner, and cause of death of control group.

Subject	DSM-IV diagnoses	Manner of death	Cause of death
1	None	Natural	Pulmonary embolism
2	None	Accidental	Peritonitis
3	None	Natural	Pulmonary embolism
4	None	Accidental	Trauma
5	None	Natural	Pulmonary embolism
6	None	Natural	Congestive heart failure
7	None	Undetermined	Undetermined
8	None	Natural	ASCVD
9	None	Natural	Cardiac tamponade
10	None	Natural	ASCVD
11	None	Natural	ASCVD
12	None	Natural	Pulmonary embolism
13	None	Natural	ASCVD
14	None	Natural	Acute endocarditis
15	None	Natural	Pulmonary embolism
16	None	Natural	Interstitial myocardial fibrosis
17	None	Natural	Myocardial infarct
18	None	Accidental	Trauma
19	None	Natural	ASCVD
20	None	Natural	ASCVD
21	None	Accidental	Drowning
22	None	Accidental	Trauma

ASCVD: atherosclerotic cardiovascular disease

Supplementary Table 7: Comorbid diagnoses, manner, and cause of death of PTSD group.

Subject	DSM-IV diagnoses	Manner of death	Cause of death
1	PTSD; ADR; ODC	Accidental	Drug overdose
2	PTSD; ADR	Natural	Airway obstruction
3	PTSD	Natural	ASCVD
4	PTSD; ODC	Accidental	Drug overdose
5	PTSD; ADC; OAC	Natural	ASCVD
6	PTSD; ADC	Accidental	Trauma
7	PTSD	Suicide	Incised wounds
8	PTSD; ODC; ODR	Accidental	Drug overdose
9	PTSD; ADC; ODC	Natural	ASCVD
10	PTSD; ADC	Natural	ASCVD
11	PTSD; AAC	Accidental	Trauma
12	PTSD; ADC; ODR; OAR	Natural	Cardiomegaly
13	PTSD; AAR	Natural	ASCVD
14	PTSD; AAR	Natural	Arrhythmogenic ventricular dysplasia
15	PTSD; ADR; ODR	Natural	Pulmonary embolism
16	PTSD; ADR; OAR	Undetermined	Drug overdose
17	PTSD; ADC; OAC; OAR	Accidental	Trauma
18	PTSD; AAR; ODR	Natural	Valvular heart disease
19	PTSD; ADC; ODC; OAR	Natural	ASCVD
20	PTSD; ADC; ODR; OAC	Accidental	Pulmonary embolism
21	PTSD; ADC	Natural	Complications from alcoholism
22	PTSD	Suicide	Drug overdose

AAC: alcohol abuse (current at time of death); AAR: alcohol abuse (in remission at time of death); ADC: alcohol dependence (current at time of death); ADR: alcohol dependence (in remission at time of death); ASCVD: atherosclerotic cardiovascular disease; OAC: other substance abuse (current at time of death); OAR: other substance abuse (remission at time of death); ODC: other substance dependence (current at time of death); ODR: other substance dependence (in remission at time of death)

Supplementary Methods

[^{11}C]PBR28 was labeled either with [^{11}C]methyl triflate or [^{11}C]methyl iodide synthesized using the TRACERLab™ FxC automated synthesis module (GE Medical Systems) or the AutoLoop™ (Bioscan Inc., Washington, D.C.) module, respectively, with [^{11}C]methyl iodide generation achieved via TRACERLab™ Fx-MeI. High-performance liquid chromatography (HPLC) coupled to a gamma detector were used to determine [^{11}C]PBR28 chemical and radiochemical purity. [^{11}C]PBR28 specific activity was determined from counting of an aliquot in a dose calibrator and from the mass measured by HPLC compared to a cold standard calibration curve. Identity of [^{11}C]PBR28 was confirmed by co-injection of cold PBR28.

Supplementary Materials Appendix:

Traumatic Stress Brain Research Group

Matthew J. Friedman MD, PhD [1,2] (Director), Victor E. Alvarez MD [1,3,4], David Benedek MD [9], Christopher Brady PhD [1,3,4], Dianne Cruz MS [7], David A Davis PhD [10], Ronald S. Duman PhD [1,5,6], Matthew J. Girgenti [5,6] PhD, Paul E. Holtzheimer MD [1,2], Bertrand R. D. Huber MD [1,3,4], Terence M. Keane PhD [1,3,4], Neil Kowell MD [3,4], John H. Krystal MD [1,5,6], Mark W. Logue PhD [1,3,4], Ann McKee MD [3,4], Brian Marx PhD [1,3,4], Deborah Mash PhD [14], Mark W. Miller PhD [1,3,4], William K. Scott PhD [10], Thor Stein MD [3,4], PhD, Robert Ursano MD [9], Douglas E. Williamson PhD [7,8], Erika J. Wolf PhD [1,3,4], Keith A. Young PhD [11,12,13]

1. National Center for PTSD
2. Department of Psychiatry, Geisel School of Medicine at Dartmouth
3. Departments of Psychiatry, Neurology, Biomedical Genetics, Biostatistics and/or Pathology & Laboratory Science, Boston University
4. VA Boston Healthcare System
5. Department of Psychiatry, Yale University School of Medicine
6. VA Connecticut Healthcare System
7. Department of Psychiatry & Behavioral Sciences, Duke University Medical Center
8. Durham VA Healthcare System
9. Department of Psychiatry, Uniformed Services University of Health Sciences
10. Departments of Human Genetics, Public Health Sciences and/or Neurology, University of Miami Miller School of Medicine
11. Central Texas Veterans Health Care System
12. VISN17 Center of Excellence for Research on Returning War Veterans
13. Baylor Scott & White and Texas A&M COM Psychiatry
14. Department of Biomedical Sciences, Nova Southeastern University
