# Supplementary material for: PTSD is associated with neuroimmune suppression: evidence from PET imaging and postmortem transcriptomic studies
Source: Nat Commun. 2020 May 12;11:2360. doi: 10.1038/s41467-020-15930-5 (PMC7217830; doi:10.1038/s41467-020-15930-5)
Supplement: Supplementary file 2 — Reporting Summary [file 41467_2020_15930_MOESM2_ESM.pdf]

nature research

Corresponding author(s): Kelly Cosgrove

Last updated by author(s): 2/1/2020

Reporting Summary

Nature Research wishes to improve the reproducibility of the work that we publish. This form provides structure for consistency and transparency in reporting. For further information on Nature Research policies, see Authors & Referees and the Editorial Policy Checklist.

Statistics

For all statistical analyses, confirm that the following items are present in the figure legend, table legend, main text, or Methods section.

n/a	Confirmed	
☐	☒	The exact sample size (*n*) for each experimental group/condition, given as a discrete number and unit of measurement
☒	☐	A statement on whether measurements were taken from distinct samples or whether the same sample was measured repeatedly
☐	☒	The statistical test(s) used AND whether they are one- or two-sided<br>*Only common tests should be described solely by name; describe more complex techniques in the Methods section.*
☐	☒	A description of all covariates tested
☐	☒	A description of any assumptions or corrections, such as tests of normality and adjustment for multiple comparisons
☐	☒	A full description of the statistical parameters including central tendency (e.g. means) or other basic estimates (e.g. regression coefficient) AND variation (e.g. standard deviation) or associated estimates of uncertainty (e.g. confidence intervals)
☐	☒	For null hypothesis testing, the test statistic (e.g. *F*, *t*, *r*) with confidence intervals, effect sizes, degrees of freedom and *P* value noted<br>*Give P values as exact values whenever suitable.*
☒	☐	For Bayesian analysis, information on the choice of priors and Markov chain Monte Carlo settings
☒	☐	For hierarchical and complex designs, identification of the appropriate level for tests and full reporting of outcomes
☐	☒	Estimates of effect sizes (e.g. Cohen's *d*, Pearson's *r*), indicating how they were calculated

Our web collection on statistics for biologists contains articles on many of the points above.

Software and code

Policy information about availability of computer code

Data collection	PET data were reconstructed using the MOLAR algorithm described previously in Carson et al., 2003 and cited in the manuscript. In-house scripts IDL (version 8.0) were used for skull segmentation of individual structural MR and pre-processing of PET data—including frame-by-frame motion correction, generation of summed images, co-registration to MR, generation of regional time activity curves. These are proprietary, though specific parts may be made available upon reasonable request.
Data analysis	In-house IDL scripts were used for modeling of regional time activity curves to estimate outcome parameters and are proprietary, though specific parts may be made available upon reasonable request. Custom scripts in R (version 3.4.1) were used for data visualization and statistical analysis in RStudio version 1.0.153 and are available upon reasonable request. Graphpad Prism v7 was used for visualization and statistical analysis of postmortem gene expression data.

For manuscripts utilizing custom algorithms or software that are central to the research but not yet described in published literature, software must be made available to editors/reviewers. We strongly encourage code deposition in a community repository (e.g. GitHub). See the Nature Research guidelines for submitting code & software for further information.

Data

Policy information about availability of data

All manuscripts must include a data availability statement. This statement should provide the following information, where applicable:

- Accession codes, unique identifiers, or web links for publicly available datasets
- A list of figures that have associated raw data
- A description of any restrictions on data availability

Due to the sensitive nature of human participant information, data are available upon reasonable request by contacting the corresponding author. Minimal, de-identified source data for figures 1-4 and supplementary figures 1-4 are available as a Source Data File.

October 2018

Field-specific reporting

Please select the one below that is the best fit for your research. If you are not sure, read the appropriate sections before making your selection.

☐ Life sciences ☒ Behavioural & social sciences ☐ Ecological, evolutionary & environmental sciences

For a reference copy of the document with all sections, see nature.com/documents/nr-reporting-summary-flat.pdf

Behavioural & social sciences study design

All studies must disclose on these points even when the disclosure is negative.

Study description	Quantitative cross-sectional study comparing a PTSD group (n=23) and a control group without PTSD (n=26), and comparing availability of TSPO measured by PET with PTSD symptoms and peripheral inflammatory markers across the PTSD group
Research sample	Individuals from the Greater New Haven area (age: 35 +/- 11 years, sex: 18 F, 31 M) with no trauma (n=10), history of civilian/military trauma without PTSD (n=16) or with PTSD (n=23) were chosen to be representative of trauma prevalence (80%) and ethnic breakdown representative of urban areas throughout the greater US, but a PTSD sample size exceeding the prevalence of PTSD in the US to allow for greater power of group-wise comparisons between PTSD and non-PTSD controls
Sampling strategy	Convenience sampling was used with an effort to match PTSD and control groups for demographic variables including age, sex, BMI, and ethnicity. Power analyses showed that samples of 23 individuals in each group would be sufficient to detect a small effect size of d = 0.3 with 80% at a significance threshold of alpha = 0.05.
Data collection	Participants were always accompanied by at least one clinical research assistant, who was not blinded to PTSD vs. control group membership, and at times one or more imaging technicians and members of nursing staff. The High Resolution Research Tomograph (HRRT; Siemens, Medical Solutions, Knoxville, TN, USA) was used to collect PET data and a 3.0-Tesla Siemens Prisma Fit Scanner (Siemens, Medical Solutions, Malvern, PA, USA) was used to collect structural MRI data. Radioactivity in blood samples taken during PET scan was measured using the automated blood samples (PBS-101, Veenstra Instruments, Joure, Netherlands) or the cross-calibrated well counter (1480 Wizard, Perkin-Elmer, Waltham, MA, USA), and free fraction of radiotracer in plasma was measured using an ultrafiltration device (Millipore Centrifree micropartition device 4104, Billerica, MA, USA). Symptom severity information was collected using pen on paper forms. Blood samples at intake or on scan-day were collected by trained registered nursing staff at Yale PET Center and analyzed for CRP using hs-CRP Wide Range Reagent (Cliniqa Corporation, San Marcos, CA, USA) or ultrasensitive CRP (Graham Massey Analytical Labs, Shelton, CT, USA) or for cytokines using MILLIPLEX panel assay (MilliporeSigma, Burlington, MA, USA).
Timing	Data were collected continuously from participants from 2011 to 2019.
Data exclusions	[11C]PBR28 VT values exceeding mean ± 3SD within rs6971 genotype groupings were pre-defined as outliers. Data from two individuals (1 control, 1 PTSD) were accordingly excluded due to regional [11C]PBR28 VT values that were 3.9 ± 0.6 SD above the mean within the HAB group, after confirming absence of technical issues in imaging and input function measurement, and absence of outlying values for free fraction, that may have contributed to outlying values.
Non-participation	No participants dropped out from the study.
Randomization	randomization was not relevant to the study due to no treatment or experimental condition for either group

Reporting for specific materials, systems and methods

We require information from authors about some types of materials, experimental systems and methods used in many studies. Here, indicate whether each material, system or method listed is relevant to your study. If you are not sure if a list item applies to your research, read the appropriate section before selecting a response.

Materials & experimental systems

n/a	Involved in the study	
☒	☐	Antibodies
☒	☐	Eukaryotic cell lines
☒	☐	Palaeontology
☒	☐	Animals and other organisms
☐	☒	Human research participants
☒	☐	Clinical data

Methods

n/a	Involved in the study	
☒	☐	ChIP-seq
☒	☐	Flow cytometry
☐	☒	MRI-based neuroimaging

Human research participants

Policy information about studies involving human research participants

Population characteristics	See above
Recruitment	Forty-nine individuals participated in this study and were recruited from 2011 to present via advertisements in public forums or through clinician referral. Given the requirements that participants be medically fairly healthy and be able to participate in several hours of procedures on scan days, it is less likely that the PTSD group, in particular, has representation from individuals with extremely severe PTSD, as this would likely preclude participation in the full proceedings of research study. Thus our study may be biased towards underestimating the degree of neuroimmune deficiency that might be associated with even higher PTSD or severity of comorbid medical conditions. Additionally, a self-selection bias of individuals able to participate in studies conducted on weekdays and therefore less likely to be employed full-time might either increase chronic stress associated with lack of financial security/stability and bias our study towards being over-representative of stress related to low SES and financial insecurity.
Ethics oversight	Yale University Institutional Human Investigation Committee and Yale New Haven Hospital Radiation Safety Committee

Note that full information on the approval of the study protocol must also be provided in the manuscript.

Magnetic resonance imaging

Experimental design

Design type	structural
Design specifications	N/A
Behavioral performance measures	N/A

Acquisition

Imaging type(s)	structural
Field strength	3
Sequence & imaging parameters	T1, sagittal gradient-echo (MPRAGE) sequence; EPI; FoV = 256 mm; matrix size = 256 x 256; slice thickness = 1mm without gap, 176 sagittal slices; voxel size = 1 x 1 x 1 mm3 ; TE = 2.26 ms, TR = 2530 ms, flip angle = 7°)
Area of acquisition	whole brain

Diffusion MRI ☐ Used ☒ Not used

Preprocessing

Preprocessing software	MATLAB SPM, skull segmentation
Normalization	non-linear transformation to template MR image
Normalization template	Montreal Neuroscience Institute, Colin 27 Average Brain 1998 (Holmes et al., 1998)
Noise and artifact removal	N/A
Volume censoring	N/A

Statistical modeling & inference

Model type and settings	Individual's structural T1 image was only used for registration of MNI template automatic-anatomical labeled regions of interest to the individual's PET image. No statistical analysis was conducted on the structural MRI data itself.
Effect(s) tested	N/A

Specify type of analysis: ☐ Whole brain ☐ ROI-based ☒ Both

Anatomical location(s)	Automatic anatomical labeling atlas (Tzourio-Mazoyer et al., 2002) was used as cited in manuscript
Statistic type for inference (See Eklund et al. 2016)	N/A
Correction	N/A

Models & analysis

n/a	Involved in the study
☒	☐ Functional and/or effective connectivity
☒	☐ Graph analysis
☒	☐ Multivariate modeling or predictive analysis
